# Does the length matter in acute appendicitis for the perforation risk?: A retrospective cohort study

**DOI:** 10.1097/MD.0000000000032001

**Published:** 2022-12-02

**Authors:** Cengiz Dibekoğlu

**Affiliations:** a Department of General Surgery, Demiroglu Bilim University, İstanbul, Turkey.

**Keywords:** appendicitis, hospital delay, increasing age, perforated appendicitis, weekend effect

## Abstract

It has been hypothesized that short appendices increase intraluminal pressure more rapidly and perforate more frequently than long appendices. Two hundred seventy-seven adult patients were retrospectively analyzed and underwent an appendectomy between January 2015 and August 2021. Data extracted from patient records included sex, age, time from admission to operation (hospital interval), weekday or weekend operation time, and operative and pathological findings. Operation was performed on 273 patients, of whom 178 (65.2%) were male and 95 (34.8%) female. The mean age of the male patients was 31.7 ± 08 (range 18–67), and that of the female patients was 38.9 ± 1.4 (range 18–78). Perforation was observed in 57 (20.9%) patients. Sex was not a factor in the development of perforation (*P* = .95). The mean age of the patients with and without perforation was 40.1 ± 2.2 and 32.7 ± 0.7, respectively. The perforation rate increased with age (*P* = .003). Appendix lengths were similar in both groups, and length was not a factor in the development of perforation (*P* = .83). This study found that the development of perforated appendicitis is not related to the length of the appendix. The risk of perforation increased with age.

## 1. Introduction

Acute appendicitis is a common cause of acute abdomen with an estimated risk of 7% to 8% worldwide.^[1]^ Despite recent improvements in the medical management of selected patients,^[2]^ emergency surgery is the accepted standard of care for appendicitis. Perforated appendicitis is associated with increased morbidity and mortality when compared to non-perforated acute appendicitis. The mortality risk in acute but not gangrenous appendicitis is <0.1% but increases to 0.6% in the gangrenous type. When appendicitis is perforated, the mortality rate increases to 5%.^[3]^

The traditional disease model of acute appendicitis was described in the early 20^th^ century and proposed a progressive inflammatory process triggered by luminal obstruction of the appendix and an increase in intraluminal pressure. It ends with perforation due to ischemia and infection.^[4]^

Although the length of the appendix has been reported in the literature to range from 1 to 20 cm, its average length is 7 to 8 cm. The distance between the root of the appendix, which is attached to the cecum, and its tip is defined as the anatomical length of the appendix.^[5]^

Thus, it was hypothesized that intraluminal pressure rises faster, and perforation rates are higher in shorter appendages than in long appendages.

Therefore, this study was designed to investigate perforation risk based on appendix length. Although one study evaluated the relationship between appendix length and the development of appendicitis using computed tomography scan measurements,^[6]^ any study in the English literature evaluating the relationship between perforation and appendix length could not be found. The study presented here is also unique in that it was conducted by measuring pathological specimens and evaluating perforations. As a result, this study is possibly the first in the literature to examine the association between appendix length and perforation rate.

## 2. Patients and methods

### 2.1. Patients

This retrospective analysis included 277 adult patients who underwent appendectomy for acute appendicitis at hospital between January 2015 and August 2021. Ethical approval for this study was obtained from the institutional review board (Ethical ID:21.05.2021/94). Preoperative and postoperative data extracted from patient records included sex, age, time from admission to the operation (hospital interval), laboratory and radiological findings used for diagnosis, weekday or weekend operation time, and operative and pathological findings. Appendix length was defined as the distance of the specimen from the base to the tip measured during macroscopic examination in the pathology department and was determined in centimeters.

According to the pathological examination results, 2 patients with neuroendocrine tumors and 2 with plastron appendicitis who did not undergo appendectomy were excluded from the study. In total, 273 patients were included in this study.

Acute appendicitis was diagnosed based on physical examination, complete blood count, serum biochemical analysis, and abdominal ultrasonography. If the appendix was not adequately visualized on ultrasonography or if there was a discrepancy between physical examination and ultrasonography, computed tomography was additionally used for diagnosis.

Patients were grouped according to the presence or absence of perforation, according to the surgical findings and pathological results.

### 2.2. Statistical results

Sex, perforation status, and operation time (weekend or weekday) were given as numbers and percentages, and continuous data as median (minimum-maximum). The chi-square and Fisher’s exact tests were used to analyze categorical variables, and an independent sample *t* test of variance was used to analyze continuous variables. Statistical significance was set at *P* < .05. All analyses were performed using SPSS software (version 21.0; SPSS Inc., Chicago, IL).

## 3. Results

A total of 273 patients underwent an operation; 178 (65.2%) were male and 95 (34.8%) were female. The mean age of male patients was 31.7 ± 08 years (range 18–67 years) and of female patients was 38.9 ± 1.4 years (range 18–78 years). Patient characteristics according to the presence or absence of perforations are shown in Table 1.

**Table 1:** Characteristics of patients according to presence or absence of perforation.

		Non- perforated group (N = 216)	Perforated group (N = 57)	*P* values
Age (mean yr)		32.7 ± 0.7	40.1 ± 2.2	.003
Gender	Male	141	37	.95
	Female	75	20	
Operation day	Weekday	167	45	.79
	Weekend	49	12	
Time elapsed from admission to surgery (mean hr)		5.1 ± 3.02	5.3 ± 0.7	.58
Appendix length (median cm)		6.8 ± 0.1	6.7 ± 0.2	.83

Perforation was detected in 57 (20.9%) patients. The presence of perforation was detected in 20.8% of male and 21.1% of female patients. Sex was not a factor in the development of perforation (*P* = .95).

The mean age of patients with and without perforation was 40.1 ± 2.2 and 32.7 ± 0.7, respectively. The perforation rate increased with age (*P* = .003).

On the weekend, 61 (22.3%) patients were operated. Perforation detection rates were 19.7% and 21.2% on weekends and weekdays, respectively. Whether the operation was performed on weekdays or weekends did not affect the perforation rate (*P* = .79).

The median time from admission to the emergency unit to surgery was 5.3 ± 0.7 hours in the perforation group and 5.1 ± 30.2 hours in the non-perforated group. The time from hospital admission to surgery was similar between groups (*P* = .58).

The median appendix length in the whole series was 6.5 cm (range, 3–12 cm). The appendix lengths were 6.7 ± 0.2 cm and 6.8 ± 0.1 cm in the groups with and without perforation. The appendix length was similar in both groups and was not a factor in the development of perforation (*P* = .83).

## 4. Discussion

Contrary to the original hypothesis, appendix length was not associated with an increased risk of perforation in the present study. In the hypothesis phase of the study, it was predicted that a shorter appendix length would lead to a more rapid increase in intraluminal pressure and easier perforation. The effect of appendix length on appendiceal perforations has not yet been studied. Therefore, it was determined whether the length had any effect. However, the study found that appendix length has no effect on the presence of a perforation. Although many studies have shown the relationship between appendiceal diameter and perforation,^[7,8]^ any studies showing the relationship between length and perforation could not be found. A meta-analysis study by Kacprzyk et al stated no relationship between appendix length and healthy or sick appendicitis.^[9]^ Although he states this, he does not comment on its relationship with perforated appendicitis. Another radiological study claimed that a longer appendix length prevents the formation of appendicitis, but did not evaluate the risk of perforation.^[6]^ Perforation discrimination was not performed in this study. Any other study examining the relationship between appendix length and perforation could not be found in the English literature. Therefore, the present study is the only study to examine this relationship.

In the present study, parameters other than the appendix length were also evaluated. One of them was the age of the patients. The mean age of the patients in the perforated group was statistically significantly higher than was in the non-perforated group. Many studies in the literature show that age increases the risk of perforation.^[10–15]^ In one study, it was shown that the perforation rate was 72% in geriatric patients.^[16]^ According to another study, delayed appendectomy over 24 hours is dangerous in older patients.^[17]^ In their study, Hanson et al showed that the perforation risk of patients over the age of 46 years increased statistically significantly when compared to younger patients.^[18]^ In the present study, the risk of perforated appendicitis was found with increased with age, similar to the literature. Thus, a direct correlation between increasing age and the risk of perforation was confirmed.

Sex was another parameter in the present study. Some studies in the literature show that sex facilitates perforation. According to these studies, male sex increases the perforation risk.^[12,13,19]^ In this study, similar to most studies, it was found that sex did not contribute to the formation of perforation.

Some studies have shown that the risk of perforation changes according to the day of the week after appendectomy. They claimed that perforation changes depending on whether the surgery is performed on weekdays or weekends. One study found that surgery performed on the weekend caused more complications than surgery performed on weekdays. Despite this, perforation information was not given in the same study, but it was claimed that mortality was higher on weekends.^[20]^ On the other hand, other studies have shown that the weekend effect is not a factor for complicated appendicitis.^[21,22]^ In this study, it was determined that weekends did not affect perforation.

This study also examined the effect of the time elapsed between hospital and operating room admission on the risk of perforation. Many studies have stated that in-hospital delay is an essential factor for perforation.^[17,23]^ In contrast, many other studies suggest that in-hospital delay does not increase the risk of perforation.^[7,11,14,24,25]^ In the present study, it was found that delay in the hospital did not increase the risk of perforation. Several similar studies have reported this result. Most studies indicate that delay in hospital admission is a risk factor, not in-hospital delay,^[4,14]^ especially over 24 to 48 hours after the onset of symptoms.^[26]^

The limitations of this study are that it was retrospective, the number of cases was relatively low, and the length of the appendix was evaluated according to the pathology report. Instead of measuring the appendix length in the pathology laboratory after storage in formaldehyde, it would have been more accurate to measure it in fresh tissue during surgery in a prospective study. Pathology specimen results usually provide the length. It would be an even more effective study if the appendix lumen volume could be calculated using length, diameter, and wall thickness measurements during the operation.

In conclusion, this study determined that the development of perforated appendicitis is unrelated to appendix length, contrary to our hypothesis. There are other unknown factors in addition to the parameters in the appendix perforation. The fact that the risk of perforation increases with age in this study supports the findings of many previous studies.

## Acknowledgment

I would like to thank Editage (www.editage.com) for English language editing.

## Author contributions

Conceptualization, data curation, formal analysis, investigation, methodology, project administration, supervision, visualization, writing—original draft, writing—review, and editing (C.D.).

**Conceptualization:** Cengiz Dibekoğlu.

**Data curation:** Cengiz Dibekoğlu.

**Formal analysis:** Cengiz Dibekoğlu.

**Investigation:** Cengiz Dibekoğlu.

**Methodology:** Cengiz Dibekoğlu.

**Software:** Cengiz Dibekoğlu.

**Supervision:** Cengiz Dibekoğlu.

**Validation:** Cengiz Dibekoğlu.

**Visualization:** Cengiz Dibekoğlu.

**Writing – original draft:** Cengiz Dibekoğlu.

**Writing – review & editing:** Cengiz Dibekoğlu.

## References

[1] van Dijk ST van Dijk AH Dijkgraaf MG. Meta-analysis of in-hospital delay before surgery as a risk factor for complications in patients with acute appendicitis. Br J Surg. 2018;105:933–45. 29902346 10.1002/bjs.10873 PMC6033184

[2] Hansson J Khorram-Manesh A Alwindawe A. A model to select patients who may benefit from antibiotic therapy as the first line treatment of acute appendicitis at high probability. J Gastrointest Surg. 2014;18:961–7. 24263678 10.1007/s11605-013-2413-0

[3] Di Saverio S Podda M De Simone B. Diagnosis and treatment of acute appendicitis: 2020 update of the WSES Jerusalem guidlines. World J Emerg Surg. 2020;15:22. 32295644 10.1186/s13017-020-00306-3 PMC7386163

[4] Papandria D Goldstein SD Rhee D. Risk of perforation increase with delay in recognition and surgery for acute appendicitis. J Surg Res. 2013;184:723–9. 23290595 10.1016/j.jss.2012.12.008 PMC4398569

[5] Mahadevan V. Anatomy of the caecum, appendix and colon. Surgery (Oxford). 2020;38:1–6.

[6] Pickhardt PJ Suhonen J Lawrence EM. Appendiceal length as an independent risk factor for acute appendicitis. Eur Radiol. 2013;23:3311–7. 23821021 10.1007/s00330-013-2948-1

[7] Yeh DD Eid AI Young KA. Multicenter study of the treatment of appendicitis in America: acute, perforated, and gangrenous (MUSTANG), an EAST multicenter study. Ann Surg. 2021;273:548–56. 31663966 10.1097/SLA.0000000000003661

[8] Bixby SD Lucey BC Soto JA. Perforated versus nonperforated acute appendicitis: accuracy of multidetector CT detection. Radiology. 2006;241:780–6. 17114626 10.1148/radiol.2413051896

[9] Kacprzyk A DroŚ J Stefura T. Variations and morphometric features of the vermiform appendix: a systematic review and meta-analysis of 114,080 subjects with clinical implications. Clin Anat. 2020;33:85–98. 31576604 10.1002/ca.23474

[10] Naderan M Babaki AE Shoar S. Risk factors for the development of complicated appendicitis in adults. Ulus Cerrahi Derg. 2016;32:37–42. 26985166 10.5152/UCD.2015.3031 PMC4771424

[11] Kulvatunyou N Zimmerman SA Joseph B. Risk factors for perforated appendicitis in the acute care surgery era-minimizing the patient’s delayed presentation factor. J Surg Res. 2019;238:113–8. 30769247 10.1016/j.jss.2019.01.031

[12] Hester CA Pickett M Abdelfattah KR. Comparison of appendectomy for perforated appendicitis with and without abscess: a national surgical quality improvement program analysis. J Surg Res. 2020;251:159–67. 32151825 10.1016/j.jss.2019.12.054

[13] Akbulut S Koç C Şahin TT. An investigation into the factors predicting acute appendicitis and perforated appendicitis. Ulus Travma Acil Cerrahi Derg. 2021;27:434–42. 34213000 10.14744/tjtes.2020.60344

[14] Drake FT Mottey NE Farrokhi ET. Time to appendectomy and risk of perforation in acute appendicitis. JAMA Surg. 2014;149:837–44. 24990687 10.1001/jamasurg.2014.77 PMC4160117

[15] Kurt E Turanli S. The effect of climate and air pollution on the development of complicated appendicitis. Pol Przegl Chir. 2022;94:33–8.36468506

[16] Vissers RJ Lennarz WB. Pitfalls in appendicitis. Emerg Med Clin North Am. 2010;28:103–18, viii. 19945601 10.1016/j.emc.2009.09.003

[17] Westfall KM Charles AG. Risk of perforation in the era of nonemergent management for acute appendicitis. Am Surg. 2019;85:1209–12. 31775960 10.1177/000313481908501124

[18] Hanson KA Jacob D Alhaj Saleh A. In-hospital perforation risk in acute appendicitis: age matters. Am J Surg. 2020;219:65–70. 31186116 10.1016/j.amjsurg.2019.05.015

[19] Lien WC Lee WC Wang HP. Male gender is a risk factor for recurrent appendicitis following nonoperative treatment. World J Surg. 2011;35:1636–42. 21573957 10.1007/s00268-011-1132-5

[20] Goldstein SD Papandria DJ Aboagye J. The “weekend effect” in pediatric surgery—increased mortality for children undergoing urgent surgery during the weekend. J Pediatr Surg. 2014;49:1087–91. 24952794 10.1016/j.jpedsurg.2014.01.001

[21] Lane RS Tashiro J Burroway BW. Weekend vs. weekday appendectomy for complicated appendicitis, effects on outcomes and operative approach. Pediatr Surg Int. 2018;34:621–8. 29626244 10.1007/s00383-018-4260-2

[22] Worni M Østbye T Gandhi M. Laparoscopic appendectomy outcomes on the weekend and during the week are no different: a national study of 151,774 patients. World J Surg. 2012;36:1527–33. 22411091 10.1007/s00268-012-1550-z

[23] Ashkenazi I Zeina AR Olsha O. In-hospital delay of surgery increases the rate of complicated appendicitis in patients presenting with short duration of symptoms: a retrospective cohort study. Eur J Trauma Emerg Surg. 2022;48:3879–86. 35211772 10.1007/s00068-022-01912-3

[24] Bhangu A Søreide K Di Saverio S. Acute appendicitis: modern understanding of pathogenesis, diagnosis, and management. Lancet. 2015;386:1278–87. 26460662 10.1016/S0140-6736(15)00275-5

[25] Fair BA Kubasiak JC Janssen I. The impact of operative timing on outcomes of appendicitis: a national surgical quality improvement project analysis. Am J Surg. 2015;209:498–502. 25557970 10.1016/j.amjsurg.2014.10.013

[26] Jiang L Liu Z Tong X. Does the time from symptom onset to surgery affect the outcomes of patients with acute appendicitis? A prospective cohort study of 255 patients. Asian J Endosc Surg. 2021;14:361–7. 32996273 10.1111/ases.12870

